# Defining the syndrome associated with congenital Zika virus infection

**DOI:** 10.2471/BLT.16.176990

**Published:** 2016-06-01

**Authors:** Anthony Costello, Tarun Dua, Pablo Duran, Metin Gülmezoglu, Olufemi T Oladapo, William Perea, João Pires, Pilar Ramon-Pardo, Nigel Rollins, Shekhar Saxena

**Affiliations:** a Department of Maternal, Newborn, Child and Adolescent Health, World Health Organization, Geneva, Switzerland.; b Department of Mental Health and Substance Abuse, World Health Organization, 20 Avenue Appia, 1211 Geneva 27, Switzerland.; c Center For Perinatology, Women and Reproductive Health, Pan American Health Organization/World Health Organization, Montevideo, Uruguay.; d Department of Reproductive Health and Research, World Health Organization, Geneva, Switzerland.; e Department of Pandemic and Epidemic Diseases, World Health Organization, Geneva, Switzerland.; f Division of Communicable Diseases and Health Security, World Health Organization Regional Office for Europe, Copenhagen, Denmark.; g Department of Communicable Diseases and Health Analysis, Pan American Health Organization/ World Health Organization, Washington, USA.

Zika virus infection in humans is usually mild or asymptomatic. However, some babies born to women infected with Zika virus have severe neurological sequelae. An unusual cluster of cases of congenital microcephaly and other neurological disorders in the WHO Region of the Americas, led to the declaration of a public health emergency of international concern by the World Health Organization (WHO) on 1 February 2016. By 5 May 2016, reports of newborns or fetuses with microcephaly or other malformations – presumably associated with Zika virus infection – have been described in the following countries and territories: Brazil (1271 cases); Cabo Verde (3 cases); Colombia (7 cases); French Polynesia (8 cases); Martinique (2 cases) and Panama (4 cases). Additional cases were also reported in Slovenia and the United States of America, in which the mothers had histories of travel to Brazil during their pregnancies.^1^

Zika virus is an intensely neurotropic virus that particularly targets neural progenitor cells but also – to a lesser extent – neuronal cells in all stages of maturity. Viral cerebritis can disrupt cerebral embryogenesis and result in microcephaly and other neurological abnormalities.^2^ Zika virus has been isolated from the brains and cerebrospinal fluid of neonates born with congenital microcephaly and identified in the placental tissue of mothers who had had clinical symptoms consistent with Zika virus infection during their pregnancies.^3^^–^^5^ The spatiotemporal association of cases of microcephaly with the Zika virus outbreak and the evidence emerging from case reports and epidemiologic studies, has led to a strong scientific consensus that Zika virus is implicated in congenital abnormalities.^6^^,^^7^

Existing evidence and unpublished data shared with WHO highlight the wider range of congenital abnormalities probably associated with the acquisition of Zika virus infection *in utero*. In addition to microcephaly, other manifestations include craniofacial disproportion, spasticity, seizures, irritability and brainstem dysfunction including feeding difficulties, ocular abnormalities and findings on neuroimaging such as calcifications, cortical disorders and ventriculomegaly.^3^^–^^6^^,^^8^^–^^10^ Similar to other infections acquired *in utero*, cases range in severity; some babies have been reported to have neurological abnormalities with a normal head circumference. Preliminary data from Colombia and Panama also suggest that the genitourinary, cardiac and digestive systems can be affected (Pilar Ramon-Pardo, unpublished data).

The range of abnormalities seen and the likely causal relationship with Zika virus infection suggest the presence of a new congenital syndrome. WHO has set in place a process for defining the spectrum of this syndrome. The process focuses on mapping and analysing the clinical manifestations encompassing the neurological, hearing, visual and other abnormalities, and neuroimaging findings. WHO will need good antenatal and postnatal histories and follow-up data, sound laboratory results, exclusion of other etiologies and analysis of imaging findings to properly delineate this syndrome. The scope of the syndrome will expand as further information and longer follow-up of affected children become available. The surveillance system that was established as part of the epidemic response to the outbreak initially called only for the reporting of microcephaly cases. This surveillance guidance has been expanded to include a spectrum of congenital malformations that could be associated with intrauterine Zika virus infection.^11^

Effective sharing of data is needed to define this syndrome. A few reports have described a wide range of abnormalities,^3^^–^^6^^,^^8^^–^^10^ but most data related to congenital manifestations of Zika infection remain unpublished. Global health organizations and research funders have committed to sharing data and results relevant to the Zika epidemic as openly as possible.^12^ Further analysis of data from cohorts of pregnant women with Zika virus infection are needed to understand all outcomes of Zika virus infection in pregnancy.

Thirty-seven countries and territories in the Region of the Americas now report mosquito-borne transmission of Zika virus and risk of sexual transmission. With such spread, it is possible that many thousands of infants will incur moderate to severe neurological disabilities. Therefore, routine surveillance systems and research protocols need to include a larger population than simply children with microcephaly. The health system response, including psychosocial services for women, babies and affected families will need to be fully resourced.

The Zika virus public health emergency is distinct because of its long-term health consequences and social impact. A coordinated approach to data sharing, surveillance and research is needed. WHO has thus started coordinating efforts to define the congenital Zika virus syndrome and issues an open invitation to all partners to join in this effort.
